# Decreases in Electrocardiographic R-Wave Amplitude and QT Interval Predict Myocardial Ischemic Infarction in Rhesus Monkeys with Left Anterior Descending Artery Ligation

**DOI:** 10.1371/journal.pone.0071876

**Published:** 2013-08-13

**Authors:** Xiaorong Sun, Jindan Cai, Xin Fan, Pengfei Han, Yuping Xie, Jianmin Chen, Ying Xiao, Y. James Kang

**Affiliations:** 1 Regenerative Medicine Research Center, West China Hospital, Sichuan University, Chengdu, Sichuan, China; 2 Department of Pharmacology and Toxicology, University of Louisville School of Medicine, Louisville, Kentucky, United States of America; University of Western Ontario, Canada

## Abstract

Clinical studies have demonstrated the predictive values of changes in electrocardiographic (ECG) parameters for the preexisting myocardial ischemic infarction. However, a simple and early predictor for the subsequent development of myocardial infarction during the ischemic phase is of significant value for the identification of ischemic patients at high risk. The present study was undertaken by using non-human primate model of myocardial ischemic infarction to fulfill this gap. Twenty male Rhesus monkeys at age of 2–3 years old were subjected to left anterior descending artery ligation. This ligation was performed at varying position along the artery so that it produced varying sizes of myocardial infarction at the late stage. The ECG recording was undertaken before the surgical procedure, at 2 h after the ligation, and 8 weeks after the surgery for each animal. The correlation of the changes in the ECG waves in the early or the late stage with the myocardial infarction size was analyzed. The R wave depression and the QT shortening in the early ischemic stage were found to have an inverse correlation with the myocardial infarction size. At the late stage, the R wave depression, the QT prolongation, the QRS score, and the ST segment elevation were all closely correlated with the developed infarction size. The poor R wave progression was identified at both the early ischemic and the late infarction stages. Therefore, the present study using non-human primate model of myocardial ischemic infarction identified the decreases in the R wave and the QT interval as early predictors of myocardial infarction. Validation of these parameters in clinical studies would greatly help identifying patients with myocardial ischemia at high risk for the subsequent development of myocardial infarction.

## Introduction

The early diagnosis and identification of high-risk patients with myocardial infarction is a practical problem in clinical setting. Improved electrocardiographic (ECG) recordings became an attractive approach for the diagnosis of myocardial infarction. Extensive efforts have been made to define criteria of alterations in different ECG waves associated with location and areas of myocardial infarction [1]–[4]. ST-segment elevation is a well recognized change in ECG recording in patients with myocardial infarction [5]. However, not all patients with myocardial infarction are associated with ST-segment elevation and not all ST segments elevation is caused by myocardial infarction [6]. Therefore, it was conventionally defined as ST-segment elevation myocardial infarction (STEMI) and non-ST-segment elevation myocardial infarction (NSTEMI). The percentage of MI cases with ST-segment elevation varies in different registries/databases and depends heavily on the age of patients included and the type of surveillance used. Among the registries, about 29% of US in-hospital patients with MI are STEMI [7], whereas 47% of European patients with MI are STEMI [8].

QT-prolongation is a parameter that can be used as a marker for myocardial infarction [9]. Both acute and chronic myocardial infarctions are associated with QT-prolongation [10], [11]. Therapeutic intervention such as thrombolytic therapy in acute myocardial infarction could reduce QT interval [12]. However, there are studies that show QT dispersion is not necessarily related to infarction size in patients with coronary artery disease [13].

The Selvester QRS score translates subtle changes in ventricular depolarization measured by the ECG into information about myocardial scar location and size [14]. This estimation of scar has been shown to have a high degree of correlation with autopsy-measured myocardial infarction size [15]. Therefore, the QRS scoring system has been utilized to estimate myocardial infarction size in clinical application [16].

R wave amplitude also changes in association with myocardial infarction. R wave amplitude decreases in patients with acute anterior wall myocardial infarction [17], but R wave amplitude increases significantly in precordial leads of the surface ECG during brief episodes of transmural ischemia [18]. Clinical studies have shown that after revascularization in patients with acute myocardial infarction, R wave amplitude in ECG increased [19], [20]. Patients with small or absent initial R waves in the anterior chest leads, resulting in QS complexes or “poor R wave progression,” could also be diagnosed as myocardial infarction [21]–[23].

Currently available parameters of the changes in ECG are of diagnostic values only for the preexisting myocardial infarction. The early prediction of myocardial tissue at high risk for myocardial infarction is of significant value for prevention or precaution for the adverse outcome under ischemic condition. A simple and quick screening procedure that can be indicative of further development of myocardial injury under ischemic condition is thus a valuable addition to currently available diagnostic procedures.

The highly human-resembling distribution of coronary arteries makes Rhesus monkeys unique substitutes for humans to study myocardial ischemic infarction [24]–[26]. It has been concluded that ligation of left anterior descending (LAD) artery results in myocardial infarction in monkeys [27], [28]. Therefore, a unique situation can be established, in which the LAD ligation can be varied to produce varying sizes of ischemic infarction in the left ventricle. The correlation of changes in the ECG parameters at different stages of myocardial ischemia and the subsequent infarction can be defined. The goal of this study was to identify an early and simple predictive surrogate in ECG changes during the ischemic phase for the subsequent development of myocardial infarction. We focused on screening QRS scores, ST-segment elevation, QT intervals, and R wave amplitude in the early prediction of myocardial infarction. Quantitative analyses identified that decreases in the R wave amplitude and the QT interval are simple and early predictors for myocardial ischemic infarction.

## Materials and Methods

### Animals and Animal Care

Male Rhesus (Macaca mulatta) monkeys, aged 2–3 years old and weighed 4.5 to 6.0 kg, were obtained from Chengdu Ping-An experimental animal breeding and research center, a Chinese government accredited non-human primate center in Sichuan province. The animals were acclimatized to the laboratory condition for a period of at least one month in the Association for Assessment and Accreditation of Laboratory Animal Care accredited facility. They were housed in individual stainless steel cages (0.8×0.9×0.78 m) in a controlled environment (at 18–28°C and 40–70% relative humidity) under controlled light-dark cycle (lights on at 8:00 and off at 20:00). Monkeys were fed completely formulated feed (two times per day, 100–150 g each time), purchased from Beijing Ke’ao Xieli Feed Co., Ltd (license number SCXK-2009-0012), and were allowed free access to drinking water produced by a reverse osmosis system. In addition, they were provided with seasonal fresh fruits including apple, banana, watermelon, and orange (100–250 g each time) three times weekly. Fruits were soaked in 0.4% disinfectant solution for 15 min followed by washing with clean water. The environment enrichment included a metal mirror attached to each cage, video watching twice a week (30 min each time) and toy playing (plastic balls and swing ring). All monkeys were handled in strict accordance with good animal practice under supervision of veterinarians, and monitored for evidence of disease and changes in attitude, appetite, or behavior suggestive of illness. Every effort was made to alleviate animal discomfort and pain by appropriate and routine use of anesthetic and/or analgesic agents. To ameliorate pain after surgery, analgesics tramadol (2 mg/kg) were injected intramuscularly once daily for 3 days. Animals were sacrificed under anesthesia (10 mg/kg ketamine and 0.2 mg/kg midazolam) at the end of the experiment. All animal procedures were approved by the Institutional Animal Care and Use Committee at the Sichuan University West China Hospital, following the guideline of the US National Institutes of Health.

### Experimental Preparation

Prior to experimental procedure, all subjects received an intramuscular injection of 10 mg/kg ketamine and 0.2 mg/kg midazolam to induce sedation. Hairs covering chest and limbs at electrode attachment sites were shaved thoroughly for better ECG recording. The standard bipolar and unipolar limb leads were recorded. Animals displaying abnormal ECG, such as tachycardia (more than 200 beats per minute), arrhythmia, and obvious ST segments deviated from the base line were excluded from this study.

### Induction of Myocardial Ischemic Infarction

All of the monkeys (n = 20) subjected to surgical procedure were intubated after anesthesia induced by intravenous infusion with fentanyl (10 µg/kg), midazolam (0.2 mg/kg), propofol (1 mg/kg), and vecuronium (0.1 mg/kg). Animals were intubated and maintained with fentanyl (5–10 µg·kg^−1^·min^−1^) and propofol (4–12 mg·kg^−1^·min^−1^) using an infusion pump. Noninvasive monitoring procedures including electrocardiography, cuff blood pressure, pulse oximetry, and capnography (Dash3000, GE, USA.) were used, and vein catheters were established.

The heart of monkeys was exposed via the left fourth intercostal thoracotomy incision (4–5 cm) in the chest wall. Two groups were divided: sham-operated control (n = 4) and myocardial infarction (MI, n = 20). The sham-operated controls were subjected to the same surgical procedure with the exception of the LAD ligation. The left anterior descending artery (LAD) was occluded for 1 min followed by a 5-min reperfusion, and this occlusion-reperfusion was repeated 3 times before the eventual ligation. The pericardium, sternum and skin were closed after the cardiac hemodynamic became stable. In preparation for recovery, analgesic tramadol (2 mg/kg) was used by intramuscular injection to alleviate suffering. The endotracheal tube was retracted after the spontaneous breathing was restored. The incision was covered with sterile gauze and bandage. The bandage change was performed on alternate days and sutures were removed one week after the operation.

### Electrocardiography

Monkeys received an intramuscular injection of 10 mg/kg ketamine and 0.2 mg/kg midazolam to induce anesthesia. A 12-leads ECG (MAC8000, GE, USA.) was recorded on the supine position of each monkey at the time before, immediately after the ligation (about 2 h for the entire surgical procedure), and eight weeks after the operation using pediatric electrodes at 25 mm/s paper velocity and 10 mm/mV amplitude. The chest wall of the Rhesus monkey was not wide enough to allow 6 precordial leads at the same time even with the pediatric electrodes. Therefore, the 6 precordial leads were divided into two groups; V1, V3, and V5 were recorded in one group, and V2, V4, and V6 in another group, as reported previously [9]. In each ECG recording, we measured the ST segment amplitude at the J point, QTc, R-wave amplitude, and QRS score.

R wave amplitude (millimeter) was measured as the vertical distance from the peak of the R wave to the baseline (as defined by two consecutive PR intervals). The sum of R wave amplitudes for anterior wall (V2 through V5) was calculated. Since the heart rate of Rhesus monkeys is about twice of the human, modified Selvester QRS score were used (Table 1). QRS duration measurements (milliseconds) were made horizontally along the PR interval baseline, and amplitude measurements (millimeter) were made vertically to the baseline. The sum of QRS score for anterior wall (V2 through V5) was also calculated. ST segment elevation above the baseline was measured at the J point. The magnitude of ST segment elevation in each lead showing elevation was measured over the 12 leads. The sum of leads for anterior wall (V2 through V5) showing ST segment elevation was also calculated. QT interval measurements and QTc calculations were performed automatically with manual inspection, over-read and verified by a single observer. The QTc was corrected using Bazett’s formula. QT interval was measured from the onset of the QRS complex to the end of the T wave, defined as the intersection of the isoelectric line and the T wave. In the presence of sinus arrhythmia, QTc was averaged from entire strip.

**Table 1:** QRS Scoring System.

Lead	Duration (ms)		Amplitude Ratio		Maximal Point
I	Q≥15	(1)	R/Q≤1	(1)	2
II	Q≥20	(2)			
	Q≥15	(1)			2
aVL	Q≥15	(1)	R/Q≤1	(1)	2
aVF	Q≥25	(3)	R/Q≤1	(2)	
	Q≥20	(2)			
	Q≥15	(1)	R/Q≤2	(1)	5
V1	Any Q	(1)			
	R≥25	(2)			
	R≥20	(1)	R/S≥1	(1)	4
V2	Any Q or R≤5	(1)			
	R≥30	(2)			
	R≥25	(1)	R/S≥1.5	(1)	4
V3	Any Q or R≤10	(1)			1
V4	Q≥10	(1)	R/Q or R/S≤0.5	(2)	
			R/Q or R/S≤1	(1)	3
V5	Q≥15	(1)	R/Q or R/S≤1	(2)	
			R/Q or R/S≤2	(1)	3
V6	Q≥15	(1)	R/Q or R/S≤1	(2)	
			R/Q or R/S≤3	(1)	3

### Measurement of Myocardial Infarction Size

Monkeys received an intramuscular injection of 10 mg/kg ketamine and 0.2 mg/kg midazolam to induce anesthesia and were sacrificed by intravenous injection of potassium chloride and a complete autopsy was performed. Harvested hearts were inspected grossly for visible lesions. Then the hearts were fixed in 10% formaldehyde solution and paraffin block was prepared. Thin sections were cut and stained with Masson's trichrome for microscopic examination, as reported previously [29].

The infarction size was visualized using the tissue sections stained with Masson's trichrome and captured as digital images followed by computerized analysis. The following parameters of each image were measured using Image-Pro Plus 6.0 software: the epicardial infarction length (the length of the transmural infarction region), the endocardial infarction length (the length of endocardial infarction scar surface >50% of the whole thickness of myocardium), and the epicardial and endocardial circumferences. The infarction size was calculated as [(sum of epicardial infarction lengths/sum of epicardial circumferences+sum of endocardial infarction lengths/sum of endocardial circumferences)/2]×100 [30].

### Statistical Analysis

Data are presented as mean ± SD for measurement variables. Comparison between groups was performed with the Student’s ”*t*” test. Pearson correlation coefficients were used to evaluate the relations among the ECG parameters and the myocardial infarction size. Linear regression models were used to generate formulas from these parameters to predict the infarction size. A SPSS 18.0 statistical package (SPSS, Chicago, IL) was used and significant difference was assumed when P values were <0.05.

## Results

**Figure 1:**
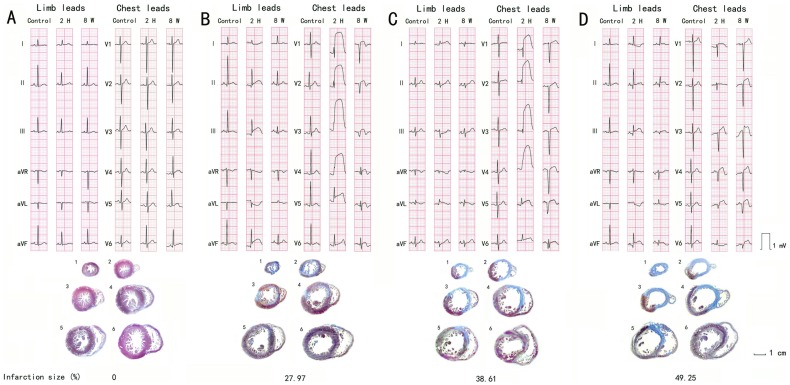
ECG recording and histopathological observation of myocardial infarction in Rhesus monkeys. (**A**) The ECG recording and histological observation obtained from a sham-operated monkey. (B) The ECG recording and histopathological observation obtained from a monkey with left ventricle infarct size of 27.97%; (**B**) 38.61%; or (**C**) 49.25%. ECG recordings, before (Control), 2 hrs after (2 H), and 8 wks after (8 W) the LAD ligation surgery, were obtained from limb leads and chest leads. Changes in the R wave amplitude and ST segment elevation are shown in the recordings. LAD = Left anterior descending.

There were 20 male Rhesus monkeys subjected to left anterior descending (LAD) artery ligation at different locations along the LAD artery, along with 4 male monkeys served as sham-operated controls. The variations in the ligation position resulted in varying sizes of myocardial ischemic infarction. The results presented in Fig. 1 show the histopathological sections of representative heart tissues with different infarction sizes along with the sham-operated controls - thoracic surgery only. The ECG recording was undertaken before the surgical procedure (Control), at 2 h after the ligation (2 H), and 8 weeks after the surgery (8 W) for each animal, as shown in Fig. 1. Thoracic surgery alone (without MI induction) in sham-operated animals did not affect ECG parameters compared to anesthetized animals without surgery, therefore, the recording obtained from sham-operated, not anesthetized animals without surgery, at each time point was presented in Fig. 1.

**Figure 2:**
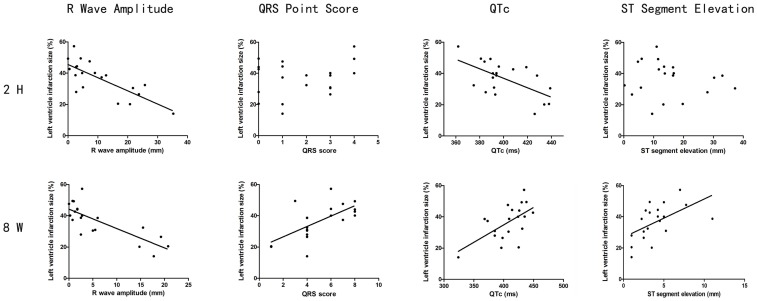
Correlation between the ECG wave changes at 2 h or 8 wks and the size (%) of myocardial infarction. The regression lines are shown in the figure. The calculated correlations as follows: R wave amplitude at 2 H: r = −0.764 (p = 0.000); at 8 W: r = −0.788 (p = 0.000). QRS score at 2 H: r = 0.237 (p = 0.315); at 8 W: r = 0.652 (p = 0.002). QTc at 2 H: r = −0.605 (p = 0.005); at 8 W: r = −0.600 (p = 0.005). ST segment elevation at 2 H: r = −0.120 (p = 0.615); at 8 W: r = 0.542 (p = 0.014).

The analyses of correlation between the ECG wave changes at 2 h or 8 wks and the size (%) of myocardial infarction were conducted for R wave amplitude, QRS score, QT interval, and ST segment elevation. The results presented in Fig. 2 show these changes at different time points. At 2 h after the LAD ligation, the R wave amplitude decreased as a function of the increase in the infarction size observed at the end of 8 wks after the surgery. Therefore, there was a good correlation between the R wave amplitude depression and the increase in the myocardial infarction size. However, the QRS score and the ST segment elevation changes during the early ischemic phase did not show correlation with the subsequent development of myocardial infarction size. Interestingly, the QT interval, as expressed as the adjusted QTc, showed a decrease as a function of the increase in the myocardial infarction size; a strong inverse correlation was detected.

At 8 wks after the LAD ligation, an inverse correlation between the R wave amplitude depression and the increase in the myocardial infarction size remained observable. However, other parameters including the QRS score and the ST segment elevation showed a positive correlation with the increase in the myocardial infarction size. In contrast to the inverse correlation between the QTc and the myocardial infarction size observed at 2 h after the surgery, a positive correlation between the QTc and the myocardial infarction size at 8 wks was observed, as shown in Fig. 2.

**Figure 3:**
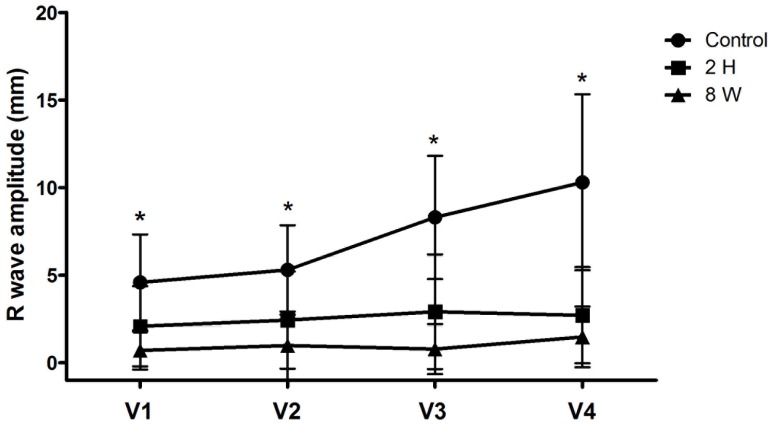
Poor R wave progression. R wave amplitude progressively increases from lead V1 to V4 monkeys before LAD ligation surgery (Control). This tendency disappeared; poor R wave progression was detected at 2 hrs (2 H) or 8 wks (8 W) after LAD ligation.

It appears that the R wave amplitude depression at both early and late time points was a consistent change. To further verify this change, we analyzed the R wave progression. As shown in Fig. 3, a poor R wave progression was observed at both 2 h and 8 wks after the LAD ligation.

## Discussion

The present study took an advantage of the non-human primate animal model as a surrogate for human cardiac disease to identify the early predictors of the ECG parameter changes for the risk of myocardial ischemic infarction. The data show that there were persistent changes in the ECG parameters that occurred both in the early and in the late stage of myocardial ischemia, such as the R wave depression. Other parameters such as the QRS score and the ST segment elevation were not changed regularly in the early stage of myocardial ischemia, but altered correlatively with the size of myocardial infarction at the late stage. An interesting change in the QT interval was that it was shortened in the early stage of myocardial ischemia, but prolonged in the subsequent development of myocardial infarction. In both cases, there was a good correlation between the change in the QT interval and the size of myocardial infarction.

Clinical studies have reported the diagnostic values of the ST segment elevation [31]–[33], the QRS score [16], [34], and the QT prolongation [12], [35] in patients with myocardial infarction. However, these parameters were only examined in the patients with preexisting myocardial infarction. The patients undergoing myocardial ischemia with high risk for myocardial infarction were not examined; such a clinical study is extremely difficult. The present study using the non-human primate model of myocardial ischemic infarction fulfilled this gap. Since the ligation position on the LAD artery can be varied, the eventual size of the myocardial infarction thus developed varies accordingly. This provides a manageable tool to analyze the correlation of the changes in the ECG parameters during the early phase of myocardial ischemia with the subsequent development of myocardial infarction, overcoming the difficulty encountered in the human clinical studies.

In the comprehensive analyses presented in this study, the R wave amplitude changes were identified to be correlative with the size of myocardial infarction at both the early and the late stage of myocardial ischemia. This finding is of significantly clinical relevance. There is at present no useful parameter of the ECG changes at the ischemic stage for the subsequent development of myocardial infarction. As discussed above, all of the clinically available parameters are predictive for the preexisting infarction or the infarction already developed. At this stage, other noninvasive procedures such as magnet resonance imaging and echocardiography are also available to detect the extent or size of the infarction in the heart. An easy and early predictor for the infarction developed in the late stage after myocardial ischemia is thus a valuable addition to the diagnostic procedures of myocardial ischemic infarction. In addition, this early prediction would help making preventive or precautious decision to minimize the risk for the subsequent development of severer myocardial infarction.

The depressed R wave amplitude has been observed in clinical studies, and suggested to be a predictor for myocardial infarction [36]. But as discussed above, it was only implicated for the application for preexisting myocardial infarction. The observation presented here shows that it is of predictive value in the early stage for the subsequent development of myocardial infarction. This would provide a new parameter for the early detection of patients at high risk for myocardial infarction. Since the R wave amplitude was obtained by the sum of the measurements from V2 to V5 leads, we analyzed the R wave progression to detect the change in the individual anterior chest lead and the trend of this change. The poor R wave progression was identified at both the early and the late stages of myocardial ischemia, therefore, further suggesting the early R wave depression as a predictor for the subsequent development of myocardial infarction.

The QT interval changes observed in the present study were interesting. At the early stage, right after myocardial ischemia, QT shortening took place and the extent correlated with the severity of ischemia and the subsequent development of myocardial infarction. In clinical observation, the short QT syndrome (SQTS) was identified to be an inherited arrhythmia disorder associated with family history of sudden cardiac death, short refractory periods, and inducible ventricular fibrillation in the absence of structural heart disease [37]–[39]. Genetic analyses identified heterogeneous forms of the SQT syndrome [40]–[42]. At present, five mutations have been associated with abnormally short QT/QTc intervals. These include three mutations linked to the gain function of the potassium channels IKr, IKs and IKl respectively in the KCNH25, KCNQ14 and KCNJ26 genes, and two mutations linked to the loss of function of the L-type calcium channels in the CACNA1c and CACNA1b genes [43]. However, the QT shortening observed in the present study resulted from the early ischemic stage, indicating that the abnormality in the coronary perfusion may also be associated with the SQT syndrome. This new potential myocardial ischemic infarction ECG marker identified here needs further evaluation. The SQT syndrome in myocardial ischemic patients was not reported. More careful and timely evaluation of myocardial ischemic patients with more sensitive equipment at the early stage may help developing new and earlier diagnostic criteria.

At the late stage, after the development of myocardial infarction, QT prolongation took place. The long QT syndromes (LQTS) were known for several decades [44]. The long QT is associated with an increased propensity to arrhythmogenic syncope, polymorphic ventricular tachy-cardia (torsades de pointes), which itself may lead to ventricular fibrillation, and sudden cardiac death. There are at least 12 different gene mutations that have been identified to associate with the LQTS (LQT1-12). Among the 12 mutations, the LQT1 and LQT2 are the most prevalent forms, associating with the loss of function of voltage-gated potassium channels (IKs and IKr) [45]. However, the QT prolongation observed in the present study would be related to the myocardial remodeling due to myocardial infarction, which has been reported in our previous studies [9]. Future studies will focus on determination of the relationship between myocardial infarction and alterations in the voltage-gated potassium channels.

In summary, the present study using non-human primate model of myocardial ischemic infarction identified the early predictors of myocardial infarction. The decreases in the R wave and the QT interval are simple measurements of the ECG changes during the early stage of myocardial ischemia. Validation of these parameters in clinical studies would greatly help identifying patients at high risk for the subsequent development of myocardial infarction.
